# Genetic adaptation of the human circadian clock to day-length latitudinal variations and relevance for affective disorders

**DOI:** 10.1186/s13059-014-0499-7

**Published:** 2014-10-30

**Authors:** Diego Forni, Uberto Pozzoli, Rachele Cagliani, Claudia Tresoldi, Giorgia Menozzi, Stefania Riva, Franca R Guerini, Giacomo P Comi, Elisabetta Bolognesi, Nereo Bresolin, Mario Clerici, Manuela Sironi

**Affiliations:** Scientific Institute IRCCS E. Medea, 23842 Bosisio Parini, LC Italy; Don C. Gnocchi Foundation ONLUS, IRCCS, 20148 Milan, Italy; Dino Ferrari Centre, Department of Physiopathology and Transplantation, Fondazione Ca’ Granda IRCCS Ospedale Maggiore Policlinico, University of Milan, 20122 Milan, Italy; Department of Physiopathology and Transplantation, University of Milan, 20090 Milan, Italy

## Abstract

**Background:**

The temporal coordination of biological processes into daily cycles is a common feature of most living organisms. In humans, disruption of circadian rhythms is commonly observed in psychiatric diseases, including schizophrenia, bipolar disorder, depression and autism. Light therapy is the most effective treatment for seasonal affective disorder and circadian-related treatments sustain antidepressant response in bipolar disorder patients. Day/night cycles represent a major circadian synchronizing signal and vary widely with latitude.

**Results:**

We apply a geographically explicit model to show that out-of-Africa migration, which led humans to occupy a wide latitudinal area, affected the evolutionary history of circadian regulatory genes. The SNPs we identify using this model display consistent signals of natural selection using tests based on population genetic differentiation and haplotype homozygosity. Signals of natural selection driven by annual photoperiod variation are detected for schizophrenia, bipolar disorder, and restless leg syndrome risk variants, in line with the circadian component of these conditions.

**Conclusions:**

Our results suggest that human populations adapted to life at different latitudes by tuning their circadian clock systems. This process also involves risk variants for neuropsychiatric conditions, suggesting possible genetic modulators for chronotherapies and candidates for interaction analysis with photoperiod-related environmental variables, such as season of birth, country of residence, shift-work or lifestyle habits.

**Electronic supplementary material:**

The online version of this article (doi:10.1186/s13059-014-0499-7) contains supplementary material, which is available to authorized users.

## Background

The temporal coordination of biological processes into daily cycles is a common feature of most living organisms. Although circadian cycles are determined by the presence of an internal cell-autonomous clock, they are synchronized (entrained) by environmental cues, most importantly visible light and external temperature [1].

In mammals, the suprachiasmatic nucleus (SCN) represents the central circadian pacemaker. SCN neurons sustain cell-autonomous cycles and, through the retinohypothalamic tract, receive inputs from melanopsin-expressing photosensitive retinal ganglion cells (RGC). Thus, although many peripheral tissues display autonomous circadian oscillations, the SCN hierarchically coordinates internal rhythms by providing a link with the external environment [2].

At the molecular level, the mammalian core circadian circuit involves the CLOCK, ARNTL (also known as BMAL1) and NPAS2 transcription factors, which activate the transcription of cryptochrome (CRY1 and CRY2), period (PER1, PER2 and PER3) and other clock-controlled genes [3]. CRY/PER heterodimers translocate back into the nucleus and inhibit their own transcription by acting on the CLOCK/ARNTL complex. The degradation of CRY and PER relieves the inhibition and initiates a new cycle [3].

Detrimental effects for health and fitness accompany alterations of circadian rhythms, due either to genetic defects or to changes in external variables that function as entrainment cues [4,5]. Notably, disruption of circadian rhythms is a common feature of human psychiatric diseases including schizophrenia (SCZ), bipolar disorder (BPD) and autism, with seasonal affective disorder (SAD) being a common condition characterized by the occurrence of depressive symptoms during short winter days [2]. Light therapy is the most effective treatment for SAD [6] and circadian-related treatments (sleep deprivation and bright light) sustain antidepressant response in patients with BPD [7].

These observations, together with the notion that all species, from bacteria to plants and animals, have evolved circadian timing systems [1], suggest that genetic adaptations to seasonal variations in day length (photoperiod) are widespread. In animals, signatures of latitude-driven natural selection at circadian genes have been described for *Drosophila* [8], birds [9,10] and fishes [11].

Anatomically modern humans appeared in East Africa about 200,000 years ago, with archeological evidence placing the origin of our species in the Awash Valley of Ethiopia [12], a region immediately north of the equator. Equatorial regions are characterized by roughly 12 h/12 h day/night cycles; thus, the annual minimum and maximum photoperiod are almost identical (that is, day length tends to be constant throughout the year). Out-of-Africa migration led humans to occupy a wide latitudinal area where seasonal variation in photoperiod can be wide. We tested the hypothesis that seasonal photoperiod variation (that is, the occurrence of day/night cycles that deviate from the 12 h/12 h pattern) acted as a selective pressure. We suggest that this process influenced adaptive evolution at circadian regulatory loci and at risk variants for psychiatric and neurologic diseases.

## Results

### Day-length variation acts as a selective pressure on genes involved in circadian rhythms

Geographically explicit models are a powerful tool to study adaptation to environmental pressures [13-20]. Thus, we applied a previously developed approach that analyzes spatial correlations between genetic variation and environmental factors [15,16] to test the hypothesis that, during out-of Africa migration, seasonal variation of annual day length acted as a selective pressure on circadian clock genes. Briefly, we analyzed genotype data from 52 human populations distributed worldwide (Human Genome Diversity Project-Centre d’Étude du Polymorphisme Humain Human Genome Diversity Cell Line Panel (HGDP-CEPH panel) [21] and determined the annual minimum and maximum photoperiod for the geographic region where each population is located (Additional file 1, Figure 1A). The difference between the maximum and minimum photoperiod (hereafter referred to as Δphotoperiod) is a measure of the deviation from the almost constant annual day length observed at equatorial regions, and is hypothesized to represent a selective pressure. For all single nucleotide polymorphisms (SNPs) in the HGDP-CEPH panel (n = 660,832) we calculated Kendall’s rank correlation between allele frequencies and Δphotoperiod. Because genetic diversity in humans is affected by demographic factors [22,23], each SNP was assigned a percentile rank in the distribution of Kendall’s correlation coefficients (τ) calculated for all SNPs with a similar (in the 1% range) minor allele frequency (MAF) calculated over all populations. This procedure was based on the assumption that demography affects the whole genome (and will be reflected in the distribution of τ values), whereas selection is a locus-specific force. Thus, SNPs that ranked high in the distribution of τ are more likely to have experienced a selective force (in addition to the demographic effect). The binning in MAF classes corrected for the fact that the power of the correlation tests was also affected by the overall SNP frequency in populations (because low MAF values result in several similar frequency values among populations - that is, several ties).

**Figure 1:**
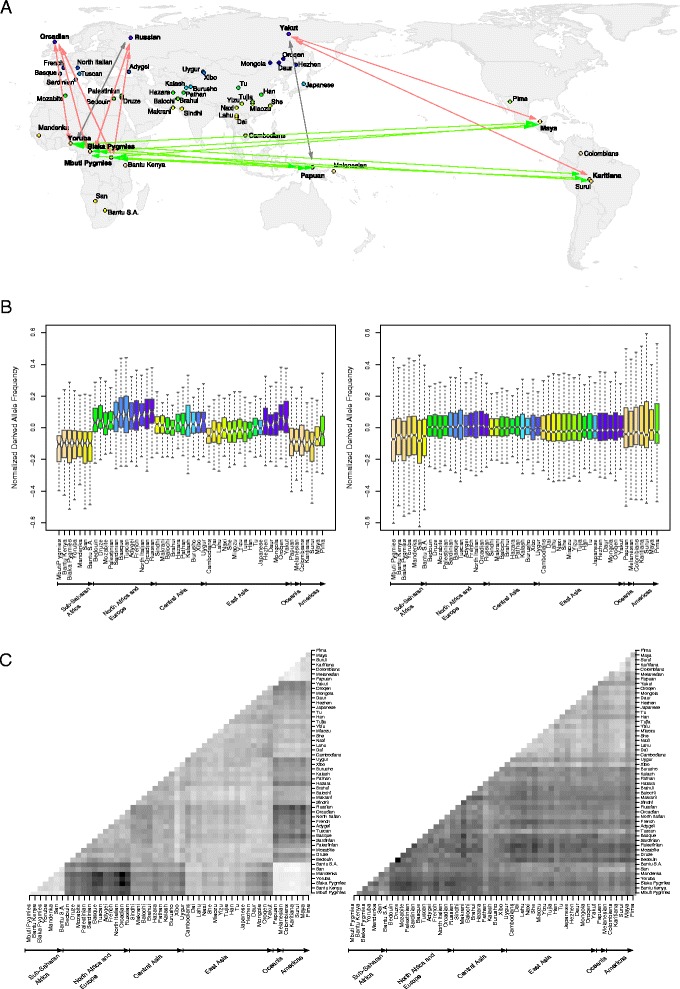
**F**_**ST**_ **(Fixation index) and allele frequency analysis. (A)** HGDP-CEPH populations are shown on a world map and color coded according to their Δphotoperiod (in topological color scale, minimum = orange, maximum = dark blue). Arrows denote populations included in F_ST_ pairwise comparisons, with red and green indicating a significant excess of variants with an F_ST_ rank >0.95 or <0.05, respectively (among the 84 SNPs in circadian rhythm genes, see text). Gray arrows denote pairwise comparisons that showed non-significant excess of high F_ST_ variants. Fisher’s exact test *P* values (see text) for F_ST_ comparisons are as follows: Orcadian-Biaka Pygmy, 0.02; Orcadian-Mbuti Pygmy, 0.05; Orcadian-Yoruba, 0.05; Russian-Biaka Pygmy, 0.03; Russian-Mbuti Pygmy, 0.03; Russian-Yoruba, 0.08; Papuan-Biaka Pygmy, <0.01; Papuan-Mbuti Pygmy, <0.01; Papuan-Yoruba, <0.01; Papuan-Yakut, 0.12; Yakut-Maya, 0.03; Yakut-Karitiana, 0.02; Maya-Biaka Pygmy, <0.01; Maya-Mbuti Pygmy, <0.01; Maya-Yoruba, <0.01; Karitiana-Biaka Pygmy, <0.01; Karitiana-Mbuti Pygmy, <0.01; Karitiana-Yoruba, <0.01. **(B)** Box-plot of normalized derived allele frequencies for the 84 SNPs significantly correlated with Δphotoperiod (left) and for 840 minor allele frequency (MAF)-matched control variants (right). Populations are color coded as in panel (A) and are grouped in six broad geographic areas; within these areas populations are ordered according to increasing Δphotoperiod. **(C)** Pairwise F_ST_ for all HGPD-CEPH populations calculated for the 84 Δphotoperiod-selected variants (left) and for 840 MAF-matched control SNPs (right). Pairwise F_ST_ percentile ranks are reported in gray scale (F_ST_ rank increasing with gray shading). Populations are ordered as in **(A)**.

We first assessed whether 12 genes that compose the core circadian pacemaker (*CLOCK*, *PER1*, *PER2*, *PER3*, *NPAS2*, *ARNTL*, *CRY1*, *CRY2*, *CSNK1E*, *CSNK1D*, *NR1D1* and *ARNTL2*) [3] carry SNPs that correlate with Δphotoperiod (Additional file 2). Specifically, we considered a variant to significantly correlate with this parameter if it displayed a Kendall’s correlation *P* value <0.05 after Bonferroni correction for the number of SNPs being analyzed (175 in the case of circadian core components) and a τ percentile rank >0.95. Seven circadian genes carried at least one variant significantly correlated with Δphotoperiod (Table 1, Additional file 2). By performing 10,000 resamplings of 12 randomly selected human genes (see Methods for details), we verified that the empirical probability of obtaining seven or more genes with at least one significant SNP amounts to 0.031, indicating that core circadian genes are more likely than expected to carry variants correlated to Δphotoperiod. Because these loci represent a small set, we next analyzed 267 genes that have been identified in a large-scale RNA interference (RNAi) screen as modifiers of circadian rhythms in U2OS cells, a human osteosarcoma cell line (Additional file 2) [24]. After removing core circadian genes (to make the two sets independent) and genes that have no SNP genotyped in the HGDP-CEPH panel, 223 RNAi hits remained; of these, 40 carried variants that significantly correlated with Δphotoperiod (Table 1, Additional file 2). Using a resampling approach as described above, we calculated that the empirical probability of observing 40 or more significant genes is 0.043.

**Table 1:** **Single nucleotide polymorphisms that correlate with Δphotoperiod**

**SNP**	**Gene**	**Kendall's correlation P value** ^**a**^	**τ rank** ^**b**^	**F**_**ST**_ **rank** ^**c**^		**LnRsb rank** ^**d**^		**Functional annotation** ^**e**^
				**Biaka-Orcadian**	**Biaka-Maya**	**CEPH**	**CHB + JPT**	
**Core circadian**								
rs11048980	*ARNTL2*	7.167 × 10^-8^	1.000	0.506	**0.000**	0.79	0.89	intronic
rs6811520	*CLOCK*	1.444 × 10^-5^	0.978	0.831	0.466	0.84	0.87	intronic
rs11038695	*CRY2*	1.626 × 10^-4^	0.957	0.624	0.247	0.64	0.33	intronic; in LD (*r* ^*2*^ = 0.93 in CEPH) with rs11038697 (within ARNTL and CLOCK binding sites)
rs4789846	*CSNK1D*	5.546 × 10^-5^	0.970	0.601	**0.000**	0.69	0.84	intronic
rs5995572	*CSNK1E*	1.053 × 10^-5^	0.984	**0.973**	0.515	0.05	0.14	intronic
rs17654772	*NPAS2*	2.549 × 10^-7^	0.998	0.615	0.377	0.84	0.43	intronic
rs4663868	*PER2*	9.462 × 10^-5^	0.962	0.286	0.219	**0.97**	0.90	intronic
**Circadian hits (RNAi screen)**								
rs2583836	*ABL1*	3.071 × 10^-7^	0.995	0.936	0.311	**0.97**	0.63	intronic
rs12713670	*ANTXR1*	4.331 × 10^-9^	1.000	0.108	0.468	0.57	0.58	intronic
rs12193789	*ASCC3*	9.351 × 10^-6^	0.994	0.371	0.166	0.94	**0.96**	intronic
rs11581556	*ATF6*	7.326 × 10^-7^	0.995	0.845	0.615	**0.96**	0.90	intronic
rs5996513	*BCR*	8.368 × 10^-6^	0.996	0.932	0.626	0.11	0.21	intronic
rs8	*CDK6*	9.581 × 10^-7^	0.998	0.800	0.299	0.82	0.85	intronic
rs2304593	*CMTM7*	5.239 × 10^-6^	0.993	0.370	**0.000**	0.16	0.34	intronic
rs2279103	*CTDP1*	6.403 × 10^-8^	0.999	0.745	0.276	0.70	0.50	missense T340M
rs657801	*DENND2D*	9.280 × 10^-6^	0.983	0.797	0.352	0.94	0.94	intronic
rs2354420	*EMP2*	6.654 × 10^-6^	0.988	0.657	0.167	0.08	0.18	intronic
rs11792480	*ENG*	1.281 × 10^-5^	0.989	**0.954**	**0.041**	0.83	0.68	intronic
rs550897	*FAM55D*	3.670 × 10^-8^	0.998	0.848	0.308	**0.99**	**0.99**	missense Y398H
rs1011814	*FGF10*	6.501 × 10^-7^	0.994	0.926	0.513	**0.99**	**1.00**	intronic
rs17679400	*FHIT*	1.3645 × 10^-7^	0.998	0.742	0.255	0.26	0.20	intronic
rs1416995	*GPR158*	2.921 × 10^-7^	0.997	**0.995**	0.973	0.04	0.29	intronic
rs12787863	*GRM5*	3.597 × 10^-7^	0.996	0.860	0.088	0.94	0.81	intronic
rs17422	*HCFC1*	1.623 × 10^-7^	0.997	0.936	**0.004**	0.84	0.81	intronic
rs11586100	*HNRNPR*	4.271 × 10^-6^	0.984	**0.968**	0.248	**1.00**	**1.00**	intronic
rs1122821	*HOMER3*	8.660 × 10^-6^	0.980	0.479	**0.013**	0.46	0.61	intronic
rs2158622	*JAZF1*	5.810 × 10^-6^	0.991	0.716	**0.000**	0.04	0.49	intronic
rs9952025	*LIPG*	1.269 × 10^-5^	0.994	0.764	0.787	0.18	0.11	intronic
rs955816	*MARCH4*	1.735 × 10^-6^	0.991	0.910	0.186	0.85	0.37	intronic; in LD (*r* ^*2*^ = 0.80 in CHB + JPT) with rs11691655 (within ARNTL and CLOCK binding sites)
rs566125	*MMP3*	1.247 × 10^-6^	0.995	0.716	0.254	**0.98**	**0.96**	intronic
rs710080	*MPG*	1.641 × 10^-7^	0.998	**0.989**	0.840	0.44	0.54	5' UTR variant
rs1053000	*PFKP*	9.234 × 10^-6^	0.988	**0.971**	0.152	**1.00**	**1.00**	3' UTR variant
rs578096	*PTGER3*	1.272 × 10^-5^	0.985	0.733	**0.000**	0.77	0.51	intronic
rs2020945	*PWP2*	5.640 × 10^-8^	0.998	**0.965**	0.780	0.62	0.50	missense D25N
rs11617401	*RAB20*	5.288 × 10^-7^	0.997	0.791	**0.000**	0.89	0.47	intronic
rs1204897	*RCC2*	7.331 × 10^-6^	0.987	0.540	0.496	0.37	0.46	intronic
rs17682132	*RCVRN*	3.072 × 10^-7^	0.998	0.943	**0.000**	**0.96**	0.33	intronic
rs492786	*SCARA3*	7.034 × 10^-6^	0.982	0.197	0.417	0.82	0.68	intronic
rs9812406	*SCHIP1*	1.487 × 10^-7^	0.998	**0.989**	0.577	0.60	0.68	intronic
rs6695715	*SEC16B*	1.113 × 10^-5^	0.982	0.247	0.123	0.44	0.78	intronic
rs2224957	*SH3GL2*	2.399 × 10^-7^	0.996	0.896	0.180	0.01	0.04	intronic
rs6081636	*SLC24A3*	5.980 × 10^-6^	0.988	0.612	0.246	0.16	0.47	intronic
rs830142	*SLC8A2*	7.264 × 10^-6^	0.993	0.325	**0.000**	0.67	0.80	intronic
rs732611	*TBC1D9*	4.321 × 10^-8^	0.999	0.943	**0.000**	0.78	0.87	intronic
rs1514685	*TPO*	5.156 × 10^-8^	0.998	0.720	0.199	0.23	0.17	intronic
rs11760463	*WDR86*	1.279 × 10^-5^	0.975	0.854	0.378	**1.00**	**0.97**	intronic
rs10227271	*WNT2*	6.406 × 10^-6^	0.983	**0.971**	0.173	0.07	0.70	intronic
**Mouse circadian/sleep disturbance**								
rs3753472	*ADORA1*	1.155 × 10^-5^	0.981	0.941	0.445	0.80	0.21	intronic
rs2830044	*APP*	5.206 × 10^-6^	0.985	0.833	0.311	0.53	0.71	intronic
rs228188	*BTBD9*	3.149 × 10^-6^	0.993	0.895	0.155	**1.00**	0.72	intronic
rs7801807	*CADPS2*	1.465 × 10^-5^	0.993	0.891	**0.000**	0.90	0.90	intronic
rs17818083	*EBF2*	2.924 × 10^-6^	0.992	0.939	**0.000**	0.11	0.24	intronic
rs809192	*FYN*	1.270 × 10^-5^	0.987	0.804	0.061	0.82	0.63	intronic
rs2134294	*HCRTR2*	5.923 × 10^-6^	0.986	0.741	0.405	**1.00**	**0.96**	intronic
rs17187747	*KCNB2*	6.700 × 10^-7^	0.994	0.652	0.349	0.33	0.54	intronic
rs12762512	*KCNMA1*	4.857 × 10^-8^	0.999	0.307	0.494	0.29	0.33	intronic
rs1943620	*NCAM1*	9.774 × 10^-9^	0.999	0.838	0.410	0.18	0.19	intronic
rs10774910	*NOS1*	1.693 × 10^-6^	0.990	0.916	0.118	**1.00**	**1.00**	intronic
rs2294678	*NOX3*	1.530 × 10^-5^	0.980	**0.950**	0.172	0.18	0.87	intronic
rs12043436	*OMA1*	2.192 × 10^-6^	0.991	0.840	0.129	**0.99**	**0.95**	intronic
rs595146	*PHLPP1*	8.637 × 10^-6^	0.999	0.877	**0.000**	0.34	0.29	intronic
rs10508958	*PRKG1*	4.490 × 10^-7^	0.996	0.873	0.193	**0.99**	0.69	intronic
rs3733553	*PRKG2*	1.212 × 10^-5^	0.980	0.868	0.823	**0.96**	**0.99**	intronic
rs752579	*RAI1*	1.023 × 10^-5^	0.981	**0.954**	0.473	**1.00**	**0.96**	intronic
rs2271733	*RAX*	1.464 × 10^-5^	0.984	0.688	0.443	0.89	0.63	missense D44E
rs10519052	*RORA*	3.667 × 10^-6^	0.991	0.448	0.234	0.68	0.68	intronic
rs968357	*RORB*	2.655 × 10^-6^	0.992	0.755	0.560	0.63	0.83	intronic
rs2236409	*TNC*	9.344 × 10^-6^	0.985	0.455	0.063	0.40	**0.96**	intronic
rs7300641	*TPH2*	1.676 × 10^-5^	0.975	0.827	0.400	0.01	0.02	intronic
rs4445877	*UBE3A*	1.849 × 10^-5^	0.974	0.939	0.607	0.90	**0.99**	intronic
**Mendelian diseases causing sleep disturbance in humans**								
rs5909187	*CDKL5*	6.275 × 10^-6^	0.987	0.550	**0.017**	0.27	0.55	intronic
rs11603330	*DHCR7*	2.848 × 10^-7^	0.995	0.806	0.479	0.75	**0.98**	intronic
rs13414769	*HDAC4*	3.134 × 10^-5^	0.969	0.573	0.264	0.94	**0.98**	intronic
rs2239464	*MECP2*	3.189 × 10^-7^	0.996	0.663	0.517	0.48	0.28	intronic
rs858953	*NRXN1*	1.226 × 10^-9^	1.000	0.780	0.254	0.83	0.49	intronic
rs8137951	*SHANK3*	3.148 × 10^-6^	0.989	0.517	0.127	0.59	0.44	intronic
rs12406072	*SLC2A1*	2.430 × 10^-5^	0.980	**0.952**	0.518	0.56	0.52	intronic
**Melanopsin signaling**								
rs308039	*GNA11*	2.387 × 10^-5^	0.972	0.568	0.079	0.06	0.29	intronic
rs4745672	*GNAQ*	4.391 × 10^-6^	0.986	0.790	0.610	**1.00**	0.92	intronic
rs2476197	*INADL*	1.834 × 10^-7^	0.998	0.943	0.252	0.92	**0.96**	intronic
rs2224361	*PLCB4*	4.222 × 10^-5^	0.991	0.426	**0.000**	0.32	0.37	intronic
rs2138004	*PRKCA*	3.025 × 10^-5^	0.971	**0.974**	0.708	0.24	0.33	intronic
rs10910030	*PRKCZ*	1.588 × 10^-8^	0.999	0.771	0.234	0.18	0.27	intronic
rs1392171	*TRPC7*	2.123 × 10^-6^	0.993	0.371	**0.000**	0.48	0.70	intronic

^a^Kendall’s correlation *P* values for the correlation between allele frequency and Δphotoperiod; ^b^percentile rank in the distribution of Kendall’s correlation coefficients (τ) calculated for minor allele frequency (MAF)-matched SNPs; ^c^F_ST_ percentile rank in the distribution of SNPs showing a similar average MAF (calculated over all populations); ^d^lnRsb percentile rank; ^e^information on ARNTL, CLOCK and CRY1 binding sites was obtained from [80]; significant values are shown in bold. CEPH, 1000 Genomes Phase I data for Utah Residents with Northern and Western European ancestry; CHB + JPT, 1000 Genomes Phase I data for Han Chinese in Beijing plus Japanese in Tokyo; RNAi, RNA interference; SNP, single nucleotide polymorphism, UTR, untranslated region.

We next analyzed genes that, when mutated or over-expressed, affect circadian rhythmicity *in vivo*. To this aim, we searched the Mouse Genome Informatics (MGI) resource for mutant strains showing circadian disturbance. Because regulation of sleep/wake cycles is a major physiological output of the circadian clock, genes that determine abnormal sleep patterns in mice were also included. After excluding core circadian genes and RNAi hits, we identified 107 loci (Additional file 2). Eighty-two of these had been included in the HGDP-CEPH panel and 23 carried variants that correlate with Δphotoperiod (Table 1). Again, these genes are more like to carry significant SNPs than expected if randomness alone were responsible (empirical *P* = 0.025).

Likewise, we analyzed the Online Mendelian Inheritance in Man (OMIM) and PhenomicDB databases to compile a list of genes that have been associated with sleep pattern disturbance in humans. Specifically, we included genes only if the phenotype (either syndromic or not) resulted from mutation and could be ascribed to a single gene. Although sleep disturbances have been observed in patients with neurodegenerative diseases (for example, Parkinson’s and Huntington’s disease), the causal genes were not considered because sleep problems in these conditions might be secondary to the general disruption of neural circuitry [2]. Also, we excluded *CSNK1D* and *PER2* (circadian core set), whose mutations cause familial advanced sleep phase syndrome, as well as *RAI1*, *BHLHE41*, *UBE3A*, and *HCRT* (responsible for Smith-Magenis/Potocki-Lupski syndromes, short sleep phenotype, Angelman syndrome and narcolepsy, respectively), which had already been included in the mouse strain gene set. Thus, we obtained a list of 11 genes, seven of which carry SNPs that correlated with Δphotoperiod (Table 1; Additional file 2). As above, this number is significantly higher than expected from random sampling (*P* = 0.0177).

Finally, we analyzed a small set of genes (n = 13) that transduce photic inputs in melanopsin-expressing RGCs; these cells sustain non-image-forming responses to light, including circadian entrainment and light-induced melatonin suppression. Again, these genes show more SNPs that correlated with Δphotoperiod than expected by chance (seven significant genes, empirical *P* = 0.0476) (Table 1, Additional file 2). We next merged together all gene sets mentioned above (core circadian, RNAi hit, mouse circadian disturbance, human sleep disturbance and melanopsin-signaling genes). Analysis of these 341 genes confirmed that they are significantly more likely than expected to carry variants that correlated with Δphotoperiod (empirical *P* =0.0335). To confirm that the results we obtained were not influenced by the SNP content of the gene sets (for example, large genes with many SNPs may be more likely to carry at least one variant that correlates with Δphotoperiod), we performed resampling analyses using SNP-matched random gene sets (Additional files 3, 4 and 5). This approach largely confirmed the results detailed above (Additional file 4).

As a further control, the same analyses were performed using environmental factors different from photoperiod variation, namely annual minimum and maximum temperature, Δtemperature (the difference between the two previous measures), and annual short-wave (UV) radiation flux. These variables are strongly correlated with Δphotoperiod, as they all depend on latitude (Kendall’s correlation coefficients with Δphotoperiod for annual minimum temperature = 0.75, maximum temperature = -0.35, Δtemperature = 0.68 and radiation flux = -0.50; all *P* values <0.0001) and may contribute to circadian entrainment [1]. In a total of 20 resampling analyses (five genes sets and four variables), only the core circadian gene set was found to display more variants than expected that correlate with Δtemperature (Additional file 6), suggesting that Δphotoperiod exerted the strongest pressure on the analyzed genes.

Thus, we analyzed five independent sets of genes involved in circadian regulation or in sleep homoeostasis and observed that they are more likely than expected by chance to carry variants showing signals of Δphotoperiod-driven selection. Notably, genes included in those five sets do not represent a full inventory of genes with a known role in circadian rhythm regulation; rather, they were selected because they could be ascribed to specific categories through dedicated searches.

**Figure 2:**
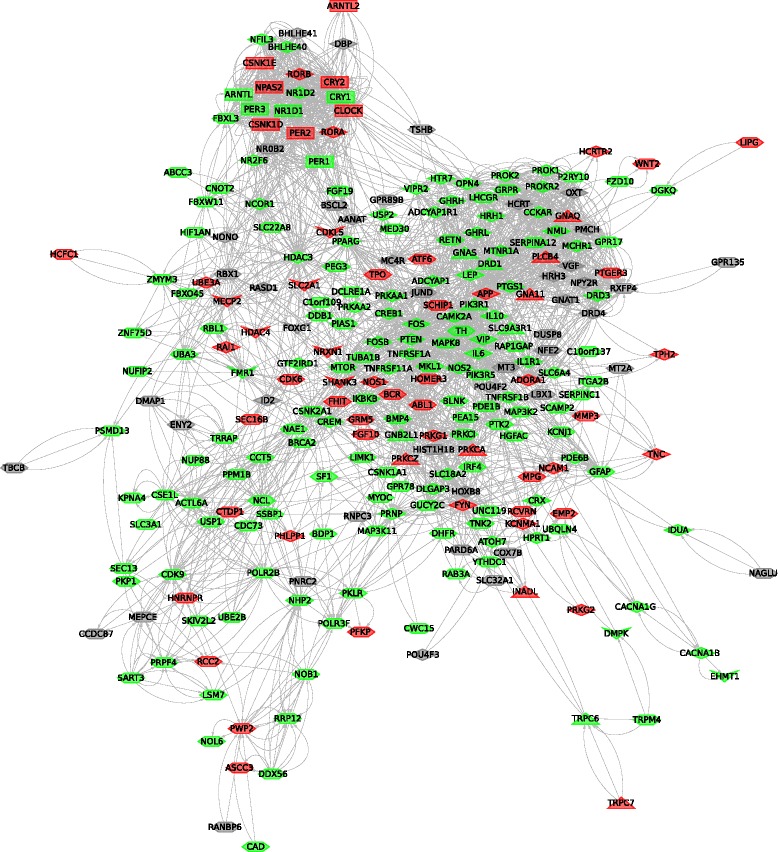
**Gene interaction network.** Nodes represent genes and edges indicate interactions. Genes are coded as follows: green, at least one single nucleotide polymorphism (SNP) in the HGDP-CEPH panel; gray, not covered in the panel; red, at least one SNP significantly correlated with Δphotoperiod. Node shapes indicate the gene set: rectangle, core circadian; hexagon, RNAi hits; diamonds, mouse model genes; triangles, melanopsin-signaling components; arrows, human phenotype genes.

In total we analyzed 406 genes in the five independent sets; 341 of these had at least one SNP in the HGDP-CEPH panel and 84 displayed signals of Δphotoperiod-driven selection. The majority (n = 273) of the full gene set (n = 406) could be included in a protein-protein interaction network (Figure 2) that comprises all core circadian and melanopsin-signaling components plus 56% of RNAi hits, 85% of circadian disturbance genes identified in mice, and 83% of human circadian phenotype loci. Two clusters of highly connected nodes are evident in the network, one that mainly includes components of the core circadian pacemaker and the other comprising neuropeptides such as hypocretin (*HCTR*), neuromedin (*NMU*), oxytocin (*OTX*), pro-melanin-concentrating hormone (*PMCH*) and their receptors. As expected, some hub nodes in the network are represented by genes involved in multiple cellular processes such as *FOS*, *APP*, *MTOR*, *PPARG* and *PTEN*.

For each of the 84 genes, we selected the SNP showing the strongest association with Δphotoperiod (based on the percentile rank of τ), obtaining a list of 84 independent variants. Four of these (4.7%) were missense substitutions (Table 1). An overview of SNP location relative to ENCODE functional elements is available as Additional file 7 for core circadian genes. Analysis of copy number variant genotypes in HGDP-CEPH and 1000 Genomes Phase I subjects [25,26] revealed no linkage disequilibrium (LD) with variants reported in Table 1.

### Natural selection signals at circadian rhythm regulatory genes

To validate the results reported above, and to gain further insight into the evolutionary history of the 84 Δphotoperiod-selected variants, we analyzed their normalized derived allele frequency (DAF): a progressive increase with Δphotoperiod was observed in most geographic areas, suggesting that selection has operated on these variants throughout human migration (Figure 1B). As expected, the normalized allele frequencies of 840 SNPs randomly selected from the HGDP-CEPH panel for having the same average MAF as selected variants (control SNPs) showed no such Δphotoperiod-dependent increase (Figure 1B).

We next reasoned that signatures of Δphotoperiod-driven selection should translate into strong genetic differentiation (fixation index, F_ST_) between population pairs that live in regions with very different Δphotoperiod (for example, Biaka Pygmies and Orcadians, Figure 1A); conversely, F_ST_ is expected to be low for geographically distant populations if they live in areas where annual variation in day length is similar (for example, Biaka Pygmies and Maya, Figure 1A). To test this hypothesis we calculated F_ST_ for the 84 variants in several pairwise comparisons (Figure 1A). To account for demographic effects, the same F_ST_ comparisons were calculated for all HGDP-CEPH SNPs and each of the 84 variants was ascribed a percentile rank in the distribution of SNPs showing a similar average MAF (calculated over all populations). In the Biaka Pygmy-Orcadian comparison, 13 SNPs showed an F_ST_ percentile rank higher than 0.95 (Table 1); this represents a three-fold enrichment over the expected (expected = 4, Fisher’s exact test, *P* = 0.019). Conversely, in the Biaka Pygmy-Maya comparison, 18 variants showed an F_ST_ below the 0.05 percentile, representing a strong enrichment over expectation (Fisher’s exact test, *P* = 0.0011, Table 1). Similar results were obtained for most additional pairwise comparisons (Figure 1A), with population pairs living in areas with very different Δphotoperiod showing an excess of SNPs with high F_ST_ values and distant populations sharing similar Δphotoperiod displaying many low-F_ST_ variants. Overall, these results confirm strong spatial signatures of natural selection at the 84 selected variants. Thus, calculation of the percentile rank of F_ST_ comparisons between all population pairs in the HGDP-CEPH panel revealed a clear signal for the 84 variants, with population differentiation increasing with Δphotoperiod across most geographic areas and low F_ST_ observed for population pairs living at similar latitudes (Figure 1C).

**Figure 3:**
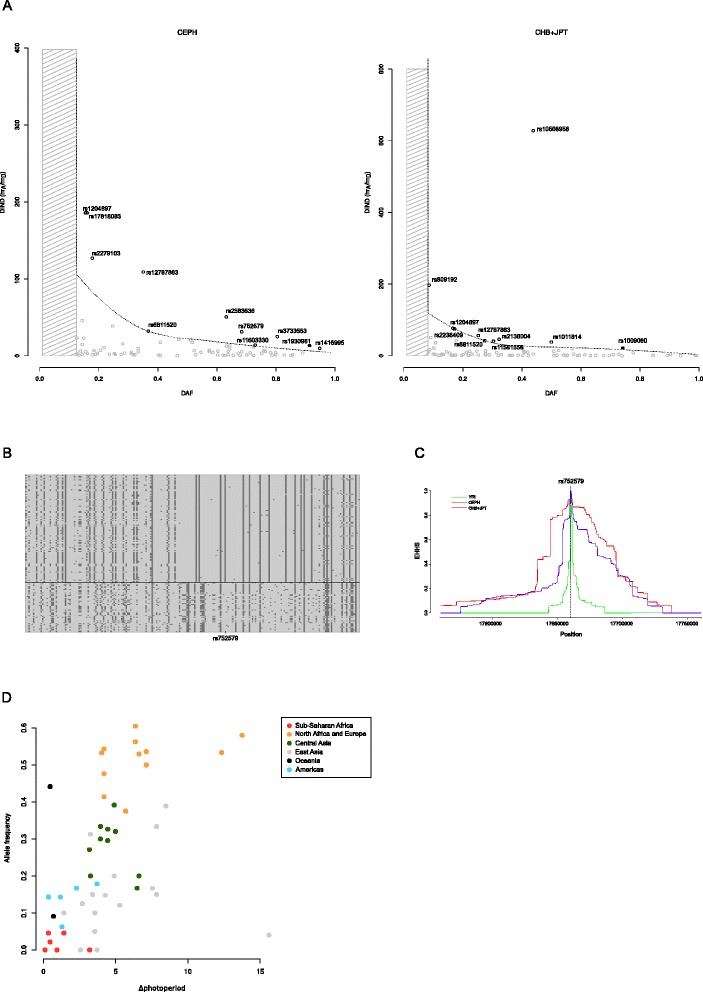
**Positive selection signatures for variants that correlate with** Δ**photoperiod. (A)** DIND test results: the dashed line represents the 95^th^ percentile of a distribution of 20,000 randomly selected HGDP-CEPH single nucleotide polymorphisms (SNPs). The gray shaded areas indicate frequency ranges where the ratio could not be calculated (see Methods). SNPs above the 95^th^ percentile are shown as black circles (open: top variants from the five gene sets; solid: disease-associated SNPs). **(B)** Schematic representation of CEPH haplotypes in the region surrounding rs752579 (in *RAI1*), selected as an example. Each line represents a haplotype, columns indicate polymorphic positions. Dark gray, derived alleles; light gray, ancestral alleles. **(C)** Extended haplotype homozygosity decay plot for rs752579. **(D)** Correlation between Δphotoperiod and allele frequency for rs752579. Populations from different broad geographic areas are coded by different colors. CEPH, 1000 Genomes Phase I data for Utah Residents with Northern and Western European ancestry; CHB + JPT, 1000 Genomes Phase I data for Han Chinese in Beijing plus Japanese in Tokyo; DIND, Derived Intra-allelic Nucleotide Diversity; EHHS, extended haplotype homozygosity of an individual SNP site.

Although different selective regimes may underlie the spatial distribution of Δphotoperiod-selected variants, it is conceivable that at least some of the signals we detected at the 84 variants are determined by selective sweeps that ensued after out-of-Africa expansion. Therefore, we tested the hypothesis that these variants are preferential targets of positive selection in non-African populations by two methods, the Derived Intra-allelic Nucleotide Diversity (DIND) [27] and lnRsb [28] tests, computed using the 1000 Genomes Phase I data for Yoruba (YRI), Utah Residents with Northern and Western European ancestry (CEPH), and Han Chinese in Beijing plus Japanese in Tokyo (CHB + JPT). DIND is based on the ratio of intra-allelic diversity associated with the ancestral and derived alleles (iπ_A_/iπ_D_) analyzed against the frequency of the derived allele [27] (Figure 3). The lnRsb test evaluates the ratio of extended haplotype homozygosity between two populations (in this case CEPH versus YRI and CHB + JPT versus YRI) [28] (Figure 3). For both tests the significance cut-off was set at *P* <0.05. DIND was calculated for CEPH and CHB + JPT for all variants with DAF >0.12 (n = 66) and >0.08 (n = 68) among the 84 we identified (see Methods). Ten SNPs had a significant DIND test in CEPH, and nine in CHB + JPT (Figure 3). In both populations the number of DIND-significant SNPs was higher than expected (Fisher’s exact test *P* = 0.038 for CEPH and *P* = 0.06 for CHB + JPT); although statistical significance is borderline in CHB + JPT, it should be noted that a three-fold excess is observed and that the sample size for Fisher’s exact test was small. Likewise, results from the lnRsb tests (calculated for all 84 variants) indicated that there are 18 significant SNPs in CEPH and 17 in CHB + JPT (Table 1), representing a significant enrichment (Fisher’s exact test, *P* = 0.0008 for CEPH and *P = *0.0015 for CHB + JPT). Signals detected using the DIND and lnRsb tests only partially overlap: this is expected as the two methods have different power to detect selection depending on the frequency of the target variant [27,28] and because the DIND can only capture selection at the derived allele.

Overall, these data suggest that variants correlated to Δphotoperiod in circadian rhythm regulatory genes represented preferential targets of positive selection during the out-of-Africa expansion of human populations.

### Genetic adaptation to photoperiod and disease susceptibility

As mentioned above, disruption of circadian rhythms is a common feature of affective disorders such as SCZ, SAD, BPD, major depressive disorder (MDD) and autism spectrum disorder (ASD). Indeed, mutations in several genes that cause sleep/wake disturbances in humans or mice (Table 1) determine a phenotype that includes ASD features (for example, *MECP2*, *SHANK3*, *NRXN1*, *RAI1*, *UBE3A*). Likewise, mice with mutations in *Clock* or *Fbxl3* (included in the RNAi hit set but showing no signatures of natural selection) display both circadian disturbances and a behavioral profile reminiscent of BPD [4]; these animals are considered models for mania [4]. Notably, genes identified in the *in vitro* RNAi screen have been implicated in susceptibility to SCZ (*SEC16B* and *CAECAM21*), ASD (*FHIT*) and MDD (*SHIP1* and *FHIT*) by genome-wide association studies (GWAS) in humans; the same holds for *ARNTL* and *RORA*, which are associated with SCZ.

Thus, we explored the possibility that risk variants for these psychiatric conditions represent targets of Δphotoperiod-driven selection. To this aim, we retrieved all GWAS SNPs associated with any trait or disease from the National Human Genome Research Institute (NHGRI) Catalog of Published Genome-Wide Association Studies [29]. We retained only variants that have been genotyped in the HGDP-CEPH panel and collapsed SNPs in LD (*r*^*2*^ > 0.4) into single loci, remaining with a list of 4,192 variants. Because more than 100 SNPs have been identified in meta-analyses of BPD and SCZ, the two diseases were considered together. Overall, our set included 128 variants associated with SCZ and/or BPD, 14 (11%) of these showing significant correlation with Δphotoperiod. By performing 10,000 resamplings we calculated that this proportion is significantly higher than that obtained for randomly selected GWAS SNPs (empirical *P* = 0.0112) and MAF-matched HGDP-CEPH variants (empirical *P* = 0.003). Thus, SCZ and/or BPD risk variants are preferential targets of natural selection driven by annual photoperiod variation. This was not the case for MDD or depression, as only two out of 50 associated SNPs significantly correlated with Δphotoperiod (Table 2). Finally, in the case of ASD, few SNPs have been associated with this condition in GWAS; thus, the LD-pruned set only contained 10 ASD variants, one of these significantly correlated with Δphotoperiod (Table 2). As for SAD, no GWAS has been performed; a variable number tandem repeat (VNTR) in the serotonin transporter gene (*SLC6A4*) promoter, which affects expression, is the only variant reproducibly associated with SAD and seasonality [30-32]. We used data from a recent analysis [33] and obtained a significant correlation between the frequency of the 14-repeat (short) allele, which predisposes to SAD [30-32], and Δphotoperiod (τ = 0.331, Kendall’s correlation *P* value = 0.00115).

**Table 2:** **Genome-wide association study single nucleotide polymorphisms that correlate with** Δ**photoperiod**

**SNP**	**Gene(s)** ^**a**^	**Disease**	**Risk allele**	**Risk allele frequency decreases with** Δ**photoperiod**	**Kendall’s correlation** ***P*** **value** ^**b**^	**τ rank** ^**c**^	**F**_**ST**_ **rank** ^**d**^		**lnRsb rank** ^**e**^		**Gene functional connection to circadian rhythm**
							**Biaka-Orcadian**	**Biaka-Maya**	**CEPH**	**CHB + JPT**	
rs1930961	*LRP5L*, *ADRBK2*	BPD	C	Yes	1.182 × 10^−6^	0.993	**0.996**	0.942	0.530	0.700	ADRBK2 (aka GRK3) phosphorylates melanopsin [34]
rs6746896	*LMAN2L*, *CNNM4*	BPD	A	Yes	8.421 × 10^−7^	0.995	0.550	0.350	0.780	0.790	*CNNM4* is an RNAi hit (the gene does not appear in Table 1 because the SNP is non-genic)
rs8099939	*GRIK5*	BPD	T	Yes	6.538 × 10^−5^	0.956	**0.968**	0.615	**0.960**	**0.980**	-
rs2286492	*FAM126A*	BPD	G	No	8.521 × 10^−5^	0.966	0.811	**0.000**	0.160	0.260	-
rs7570682	*TMEM18*, *POU3F3*	BPD	A	Yes	2.320 × 10^−4^	0.950	0.455	**0.004**	0.160	0.090	-
rs4075511	*KCNS3*, *RDH14*	BPD	A	No	7.345 × 10^−9^	0.999	0.797	0.156	0.720	0.730	-
rs7319311	*COL4A2*	BPD and SCZ	A	No	1.083 × 10^−4^	0.952	0.474	0.319	0.400	0.470	*Clock*-regulated in mouse cardiomyocytes [35]
rs1124376	*KAT2B*	BPD and SCZ	G	Yes	3.852 × 10^−4^	0.950	0.860	0.076	0.880	0.150	Directly interacts with CLOCK and NPAS2 [36]
rs1605834	*APOB*, *KLHL29*	BPD and SCZ	C	Yes	2.857 × 10^−4^	0.950	**0.966**	0.877	0.350	0.240	-
rs17002034	*MKL1*	BPD and SCZ	G	Yes	2.322 × 10^−5^	0.978	0.689	**0.000**	**0.990**	**0.990**	RNAi hit (gene does not appear among selected hits because of heavier Bonferroni correction)
rs589249	*CSF3R*, *GRIK3*	SCZ	G	Yes	4.565 × 10^−5^	0.964	**0.996**	0.624	**1.000**	**1.000**	*Grik3* expression is regulated by Clock in the mouse ventral tegmental area [37]
rs7004633	*MMP16*, *RIPK2*	SCZ	A	No	1.433 × 10^−6^	0.992	0.925	0.538	0.260	0.330	-
rs1009080	*PTPRU*, *MATN1*	SCZ	G	Yes	2.419 × 10^−6^	0.988	**0.987**	0.708	0.900	0.690	-
rs1572299	*TLR4*, *DBC1*	SCZ	A	Yes	3.901 × 10^−4^	0.951	**0.950**	0.321	**1.000**	0.200	DBC1 regulates NR1D1 stability [38]
rs892055	*RASGRP4*	Asperger	nr	na	4.268 × 10^−5^	0.964	0.603	0.585	0.610	0.420	-
rs9601248	*NDFIP2*, *SPRY2*	MDD	C	No	7.198 × 10^−5^	0.958	0.896	0.085	0.390	0.330	*SPRY2* is a direct target of ARNTL in mouse liver [39]
rs8020095	*GPHN*	Depression	A	Yes	1.273 × 10^−4^	0.950	0.944	0.504	0.620	**0.980**	*GPHN* shows rhythmic expression in mouse SCN [40]
rs12593813	*MAP2K5*	RLS	A	No	2.066 × 10^−5^	0.971	0.498	0.175	**1.000**	0.830	-

^a^One gene is reported if the variant is genic, for intergenic SNPs the two flanking protein-coding genes are listed; ^b^Kendall’s *P* values for the correlation between allele frequency and Δphotoperiod; ^c^percentile rank in the distribution of Kendall’s correlation coefficients (τ) calculated for minor allele frequency (MAF)-matched SNPs; ^d^F_ST_ percentile rank in the distribution of SNPs showing a similar average MAF (calculated over all populations); ^e^lnRsb percentile rank; significant values are shown in bold. BPD, bipolar disorder; CEPH, 1000 Genomes Phase I data for Utah Residents with Northern and Western European ancestry; CHB + JPT, 1000 Genomes Phase I data for Han Chinese in Beijing plus Japanese in Tokyo; MDD, major depressive disorder; na, not available; nr, not reported; RLS, restless leg syndrome; SCZ, schizophrenia; SNP, single nucleotide polymorphism.

Although not a psychiatric disease, restless leg syndrome (RLS) is a human condition with a strong circadian component. RLS symptoms worsen in the evening or night, and include sleep fragmentation. GWAS have identified seven unlinked SNPs, most of these mapping to four distinct genes (*MEIS1*, *BTBD9*, *PTPRD* and *MAP2K5/SKOR1*). Four of these variants have been included in the HGDP-CEPH panel and one of them significantly correlated with Δphotoperiod (rs12593813, at the *MAP2K5/SKOR1* locus) (Table 2).

As above, for all GWAS variants showing signals of Δphotoperiod-driven selection we performed the DIND and lnRsb tests. Natural selection signatures at several of these variants were detected (Table 2, Figure 3). Notably, many risk variants showing signals of Δphotoperiod-driven selection map within or close to genes with strong evidence of involvement in circadian rhythm regulation (Table 2). For example, ADRKB2 phosphorylates melanopsin [34], KAT2B directly interacts with CLOCK and NPAS2 [36], *CNNM4* and *MKL1* have been identified in the circadian RNAi screen, DBC1 regulates NR1D1 stability [38], and *GPHN* shows circadian oscillations in the mouse SCN [40].

## Discussion

We analyzed adaptation of human populations to seasonal variations in day length determined by latitude using a method based on the spatial correlation between allele frequencies and environmental variables. Our working hypothesis builds on the previously reported suitability of geographic-explicit models to study human evolution [13-17,20]; the simple conundrum whereby genes that play a role in circadian regulation should represent preferential targets of Δphotoperiod-driven selection; and the expectation that a significant fraction of variants identified using models of spatial variation in allele frequencies should display natural selection signatures detectable with different approaches. Thus, we analyzed five independent gene sets selected on the basis of distinct evidence of involvement in circadian regulation and show that they are significantly more likely to carry SNPs that correlate with Δphotoperiod than expected. Photoperiod is a function of latitude, as is the case for other environmental variables such as temperature and UV radiation flux. These other climatic factors might contribute to the evolution of the circadian genes we analyzed. Nonetheless, resampling analysis using temperature and UV radiation revealed no excess of correlated variants, with the only exception of circadian core genes with Δtemperature. This might either depend on the difficulty of disentangling the effects of correlated environmental variables or on the fact that external temperature also acts as a circadian entrainment cue [1]. Nonetheless, these analyses indicate that Δphotoperiod exerted a stronger selective pressure on these genes than temperature or UV radiation flux.

Several variants identified using geographic-explicit model display signatures of natural selection when tests based on population genetic differentiation or haplotype homozygosity are applied, supporting their adaptive role. Although these tests (in particular the DIND and lnRsb) likely detected signals that derive from so-called hard sweeps (that is, sweeps of newly arisen mutations), adaptation to seasonal variation in photoperiod possibly also occurred through balancing selection or selection on standing variation (soft sweeps), as previously suggested for other environmental variables [41-43]. The signatures left by soft sweeps are known to be more difficult to identify and typically do not originate an extended LD pattern [44,45].

Finally, we should add that variants genotyped in the HGDP-CEPH panel suffer from a moderate ascertainment bias [21], which might potentially affect our results. Nonetheless, the ascertainment scheme is common to all variants and we based most of our results on the drawing of empirical distributions, which, at least partially, correct for panel-wide biases. Also, we validated our results by the application of haplotype-based tests with the use of unbiased genotype data. Overall, the results reported here indicate that, during out-of-Africa migration, human populations adapted to life at different latitudes by tuning their circadian clock systems. In line with this observation, the length of the endogenous circadian period differs in people depending on ethnicity, and Africans show shorter periods than people with European or Asian ancestry [46].

The selective effect of photoperiod differences across geographic areas has previously been demonstrated for vertebrates and invertebrates, by studying latitudinal clines in circadian gene polymorphisms [8-11], and by dissecting rhythmic functions in animals living at extreme latitudes, where loss of ultradian patterns may be observed during periods of continuous light or darkness [1]. Indeed, the synchronization of internal clocks with environmental cues is thought to be essential for health and fitness [5]. As an example, *Clock*-mutant mice display abnormal rhythmicity of several physiological functions (for example, sleep/wake cycles, food intake and basal metabolism), show behavioral disturbances, are susceptible to diet-induced obesity, and present frequent pregnancy failures [3]. Likewise, wild-type rodents exposed to experimental jet lag show accelerated tumor growth, spatial cognitive deficits, cardiovascular problems, hastened mortality upon aging and increased body mass [5]. Similar health problems are reported to occur with high prevalence in humans who experience frequent changes in daily rhythms as a result of rotating shift work [5].

A large body of evidence suggests that circadian rhythm abnormalities determine or exacerbate mood-related disorders [4]. Alterations in sleep/wake cycles, activity, body temperature and hormonal secretions have been described in BPD, SCZ, MDD and ASD [4]. Reduced amplitude and lower expression of core circadian components have been described in fibroblasts from patients with BPD [47]. In parallel, studies in diurnal rodent species have demonstrated that prolonged exposure to a very short photoperiod results in a depression-like behavior [48,49], whereas in rats prolonged dark phase conditions administered post-natally result in increased anxiety and decreased social interaction in adult life [50]. These observations indicate a causal link between psychiatric diseases and circadian disruption and suggest that mood disorders in modern human populations might at least partially result from adaptation or mis-adaptation to latitudinal photoperiod variations. We thus tested whether risk variants for psychiatric disorders represent preferential targets of Δphotoperiod-driven selection. Results indicated that the proportion of risk variants targeted by Δphotoperiod-driven selection is significantly higher than expected for BPD and SCZ, but not for MDD, although sleep and circadian rhythm disruption represent a common feature in depressive disorders. Nonetheless, MDD differs from SCZ and BPD by showing higher prevalence and substantially lower heritability [51,52]. Fewer risk variants with smaller effect have been identified for MDD compared with SCZ and BPD. This is reflected in our study, whereby the sample size of MDD SNPs is less than half that of SCZ or BPD variants.

Additionally, recent works have suggested that the consequences of MDD on reproductive fitness might be much less severe than those of other psychiatric disorders. Large-scale studies based on historical registries have indicated that patients with SCZ have strongly reduced fecundity [53-55], whereas their siblings may have increased or decreased reproductive success depending on gender [53,54]. The overall effect calculated for affected patients and their siblings indicated that these families contribute fewer descendants to the new generation compared with families with no members affected by SCZ [53,54]. Similar results have been reported for BPD, although the decrease in fecundity seems to be less marked than that observed for SCZ [53,55]. Contrasting evidences have been reported about the fertility effect of MDD; some authors [55] indicated lower reproductive rates for patients with MDD compared with controls (however reproductive success was higher than in SCZ and BPD). Conversely, a recent analysis revealed that no reduction in fecundity is observed in patients with MDD [53]. Interestingly, siblings of patients with MDD were shown to have significantly more children.

These observations lead to a long-debated hypothesis on the adaptive role of depression [56], including SAD [57], and suggest that MDD variants are subject to selective regimes different from other psychiatric disease. As mentioned above, SAD is the best example of a mood disorder directly triggered by photoperiod changes. The observation whereby the short VNTR allele at *SCL6A4* increases with Δphotoperiod, especially across Africa and Europe [33], may not be directly supportive of the adaptive view, but at least indicates that a SAD-predisposing variant is not counter-selected at high latitudes. As for the psychiatric disease variants we identified as targets of Δphotoperiod-driven selection, the two for MDD show different patterns: the risk allele frequency increases with Δphotoperiod for *GPHN* and decreases for *NDFIP2/SPRY2*. Conversely, for the majority of SCZ or BPD risk alleles (10 in 14), a lower frequency is observed in populations showing greater Δphotoperiod. Thus, as populations expanded at higher latitudes, non-risk variants for SCZ and/or BPD might have been favored by selection to counterbalance the effects of photoperiod length variations, these latter possibly representing an environmental stress for people at risk of mental illness.

## Conclusions

Signatures of latitude-driven natural selection at circadian genes have been described for vertebrates and invertebrates [8-11]. Herein we address this same issue in humans by showing that humans adapted to seasonal variations in photoperiod.

Also, we provide a link among circadian rhythm regulators, human adaptation to latitude, and susceptibility to affective disorders. Whereas the evolutionary scenarios underlying the maintenance of affective disorder risk variants will require further exploration, the Δphotoperiod-selected risk variants we identified represent excellent candidates for interaction analysis with photoperiod-related environmental variables (for example, season of birth, country of residence, shift-work or lifestyle habits). Also, these SNPs might modulate the effect of chronotherapy, which is gaining increasing interest *per se* or in supplementation to pharmacological approaches.

## Methods

### Environmental variables

Geographic coordinates for HGDP-CEPH populations were obtained by the HGDP-CEPH website [58]. When population locations were provided as latitude ranges, the midpoint was used. For each population, the annual maximum and minimum day lengths (photoperiods) were calculated using the C++ code available as a part of the Google Wide Open Smart Home framework [59]. The annual minimum and maximum photoperiod, as well as Δphotoperiod, are monotone functions of latitude (absolute value). Thus, photoperiod variation is consistent over latitude ranges. Because we applied a rank-based correlation test, the results are robust to minor mis-specification of latitudes.

The annual minimum and maximum temperatures as well as short-wave radiation flux were retrieved from the National Centers for Environmental Prediction/National Center for Atmospheric Research database (Legates and Willmott Average, re-gridded dataset).

### HGDP-CEPH data and statistical analysis

Genotype data for the HGDP-CEPH panel derive from a previous work [21]. Eighty-six SNPs were removed as they were invariant (MAF = 0) or because they failed in all populations. Atypical or duplicated samples and pairs of close relatives were removed [60]. An SNP was ascribed to a specific gene if it was located within the transcribed region or no farther than 500 base pairs upstream of the transcription start site. MAF for any single SNP was calculated as the average over all populations. When necessary, the derived or ancestral state was determined based on the comparison with the chimpanzee, orangutan and macaque reference genomes.

Genes that determine circadian or sleep pattern disturbances in mouse were identified though the MGI website [61]. Specifically, we searched the Mammalian Phenotype Ontology using the term “circadian” and the MGI phenotypes using “circadian” and “abnormal sleep pattern” as keywords. Entries were manually inspected so that only known protein-coding genes were included (the MGI search also retrieved “heritable phenotypic markers”, with undefined genomic location); and phenotypes resulting from double gene knock-outs were not considered (as the effect cannot be unequivocally attributed to one gene). The MGI search method was preferred over conventional literature analysis to obtain an unbiased set of genes involved in mouse circadian phenotypes. Genes responsible for sleep disturbance phenotypes in humans were identified through search of the OMIM [62] and PhenomicDB [63] databases using “circadian” or “sleep disturbances” as keywords. Entries were manually inspected and genes were included only if the phenotype can be ascribed to the mutation of a single gene (for example, contiguous gene syndromes were not included); and the phenotype results from mutation. Specifically, we decided not to include polymorphism-phenotype associations, because they have been show to often lack robustness and fail to replicate in independent analyses [64]; as an example, variants in the *MAOA* gene have been associated with poor sleep quality and insomnia in two studies that analyzed few subjects and obtained partially contrasting results [65,66]). Thus, *MAOA* was not included in the study gene set.

Genes involved in melanopsin signaling were derived from a previous work [67], with the inclusion of *TRPC6*, *TRPC7* and *PLCB4* from Xue *et al*. [68].

Correlations between allele frequencies and environmental variables were calculated by Kendall’s rank correlation coefficient (τ), a non-parametric statistic used to measure the degree of correspondence between two rankings. Specifically, because all SNPs in the HGDP-CEPH panel are bi-allelic, the frequency of one allele (randomly selected) was used in the correlation test (using the other allele would simply change the sign of the correlation coefficient). To account for demographic events, each SNP is then assigned a percentile rank in the distribution of τ absolute values calculated for all SNPs having a MAF (averaged over all populations) similar (in the 1% range) to that of the SNP being analyzed [15,16].

To estimate the probability of obtaining *n* genes carrying at least one significant SNP out of a group of *m* genes, we applied a resampling approach: samples of *m* genes were randomly extracted from a list of all genes covered by at least one SNP in the HGDP-CEPH panel (number of genes =15,280) and for each sample the number of genes with at least one significant SNP was counted (Bonferroni correction was applied to each sampled SNP set). The empirical probability of obtaining *n* genes was then calculated from the distribution of counts deriving from 10,000 random samples of *m* genes. A similar resampling strategy was used for SNP sets, as mentioned in the text.

Pairwise F_ST_ was calculated using the R package hierfstat [69]. Because F_ST_ values are not independent from allele frequencies, we binned variants based on their MAF (50 classes) and calculated the 95^th^ percentile for each MAF class, as detailed in the text. The populations used for the pairwise comparisons were selected on the basis of their Δphotoperiod and for having a sufficiently large sample of individuals genotyped in the HGDP-CEPH panel (at least 13, Additional file 1). The Yakut-Orcadian, Yakut-Russian and Russian-Orcadian comparisons are not reported because F_ST_ is generally low between these populations and the 0.05 percentile could not be unequivocally determined.

### 1000 Genomes data and positive selection tests

1000 Genomes Project Phase I data were retrieved from the dedicated website [26,70]. Data refer to the following populations: CEPH (Utah residents with Northern and Western European ancestry), population code = CEU, number of individuals = 85; YRI (Yoruba in Ibadan, Nigeria), population code = YRI, number of individuals = 88; CHB (Han Chinese in Beijing, China), population code = CHB, number of individuals = 97; JPT (Japanese in Tokyo, Japan), population code = JPT, number of individuals = 89. Data for CHB and JPT were combined.

SNP genotypes were organized in a MySQL database. A set of programs was developed to retrieve genotypes from the database and to analyze them according to selected regions or populations. These programs were developed in C++ using the GeCo++ [71] and the libsequence [72] libraries.

The DIND test [27,73] was calculated for all 84 SNPs in Table 1 and for disease risk variants (Table 2); the statistical significance was derived from an empirical distribution of DIND-DAF value pairs for 20,000 SNPs randomly selected among those genotyped in the HGDP-CEPH panel. Specifically, DIND values were calculated for all SNPs using a constant number of 40 flanking variants (20 up- and downstream). The distributions of DIND-DAF pairs for CEPH and CHB + JPT were binned in DAF intervals (100 classes) and for each class the 95^th^ percentile was calculated [74]. As suggested previously [27], for values of iπ_D_ = 0 we set the DIND value to the maximum obtained over the whole dataset plus 20. For low DAF values most iπ_D_ were equal to 0 (that is, the 95th percentile could not be calculated); thus, we could not calculate the DIND test for SNPs with DAF <0.12 in CEPH and 0.08 in CHB + JPT.

The lnRsb tests and the extended haplotype homozygosity of an individual SNP site (EHHS) were calculated as previously described [28] for the 84 SNPs in Table 1 and for disease risk variant in Table 2. Specifically, lnRsb was calculated for all SNPs surrounding the selected variant in a 200 kilobase region; the statistical significance was derived from an empirical distribution of values from all SNPs in these regions. The EHHS test was calculated for the selected SNP as the marker and until it reached 0.05 in the surrounding region. The analysis was performed using the rehh R package [75].

### Analysis of disease-associated variants

A list of GWAS SNPs was obtained from the NHGRI Catalog of Published Genome-Wide Association Studies [29,76]. Among these variants we retained those that had been genotyped in the HGDP-CEPH; these were collapsed on the basis of LD, originating a set of 4,192 variants. In particular, LD between SNP pairs was calculated using SNP Annotation and Proxy Search [77] with data for Europeans (CEU); SNPs showing *r*^*2*^ ≥ 0.4 were collapsed in a single locus by randomly selecting one of the two polymorphisms. Thus, all analyzed variants, including those associated with affective disorders and RLS, show *r*^*2*^ < 0.4.

Frequency data for the *SLC4A6* VNTR were kindly provided by Dr J Murdoch as per a recent worldwide analysis [33]. Data for African Americans and European Americans were not included in the analysis as a these groups cannot be located in a specific geographic area (or are admixed).

### Gene interaction network and functional annotation

To visualize gene interactions, we used the Search Tool for the Retrieval of Interacting Genes/Proteins (STRING) utility [78]. STRING queries different interaction databases and we limited our search to the human species. The resulting interaction file was used as an input for Cytoscape 2.8.3 [79].

Information on ARNTL, CLOCK and CRY1 binding sites was obtained from previous work [80]. Data derive from chromatin immunoprecipitation sequencing experiments in U2OS cells.

### Data accession

All data used in this manuscript are publicly accessible through the HGDP-CEPH panel database (HGDP-CEPH Genome Diversity Panel Database Version 3.0, http://www.cephb.fr/hgdp/index.php) and the 1000 Genomes Project website (http://www.1000genomes.org/; 1000 Genomes Project Phase I data).

## Additional files

Additional file 1:
**Populations in the HGDP-CEPH panel with Δphotoperiod.**
Additional file 2:
**List of analyzed genes, significant SNPs and phenotype descriptions.**
Additional file 3:
**Resampling analysis with SNP number-matched gene sets.**
Additional file 4:
**Resampling results with SNP-matched random gene sets.**
Additional file 5:
**Distribution of SNP content.**
Additional file 6:
**Resampling analysis with different environmental variables.**
Additional file 7:
**Functional annotation for core circadian genes.**

## Abbreviations

ASD: autism spectrum disorder
BPD: bipolar disorder
CEPH: Utah Residents with Northern and Western European ancestry
CHB + JPT: Han Chinese in Bejing plus Japanese in Tokyo
DIND: Derived Intra-allelic Nucleotide Diversity
EHHS: extended haplotype homozygosity of an individual SNP site
GWAS: genome-wide association studies
LD: linkage disequilibrium
MAF: minor allele frequency
MDD: major depressive disorder
MGI: Mouse Genome Informatics
NHGRI: National Human Genome Research Institute
OMIM: Online Mendelian Inheritance in Man
RGC: retinal ganglion cells
RLS: restless leg syndrome
RNAi: RNA interference
SAD: seasonal affective disorder
SCN: suprachiasmatic nucleus
SCZ: schizophrenia
SNP: single nucleotide polymorphism
STRING: Search Tool for the Retrieval of Interacting Genes/Proteins
YRI: Yoruba
