# Supplementary material for: Genetic adaptation of the human circadian clock to day-length latitudinal variations and relevance for affective disorders
Source: Genome Biol. 2014 Oct 30;15(10):499. doi: 10.1186/s13059-014-0499-7 (PMC4237747; doi:10.1186/s13059-014-0499-7)
Supplement: Additional file 1: — Populations in the HGDP-CEPH panel with Δphotoperiod. [file 13059_2014_499_MOESM1_ESM.pdf]

Additional data file 1. Populations in the HGDP-CEPH panel and Δphotoperiod

Population	Country	Sampled individuals	Δphotoperiod (hours)
Bantu North East	Kenya	11	0.3472
Bantu South East	South Africa	8	3.2015
Biaka Pygmies	Central African Republic	23	0.4634
Mandenka	Senegal	22	1.4103
Mbuti Pygmies	Democratic Republic of Congo	13	0.1156
San	Namibia	5	2.5552
Yoruba	Nigeria	21	0.9317
Colombians	Colombia	7	0.3472
Karitiana	Brazil	14	1.1694
Maya	Mexico	21	2.2899
Pima	Mexico	14	3.7091
Surui	Brazil	8	1.2894
Balochi	Pakistan	24	3.9467
Brahui	Pakistan	25	3.9467
Burusho	Pakistan	25	4.99139
Hazara	Pakistan	22	4.44821
Kalash	Pakistan	23	4.89757
Makrani	Pakistan	25	3.2562
Pathan	Pakistan	23	4.44821
Sindhi	Pakistan	24	3.1833
Uygur	China	10	6.60288
Cambodians	Cambodia	10	1.4103
Dai	China	10	2.5553
Daur	China	9	7.82877
Han	China	44	4.27681
Hezhen	China	9	7.53369
Japanese	Japan	29	5.28149
Lahu	China	8	2.6909
Miaozu	China	10	3.555
Mongola	China	10	7.82877
Naxi	China	8	3.2562
Oroqen	China	9	8.46656
She	China	10	3.4041
Tu	China	10	4.89757
Tujia	China	10	3.7091
Xibo	China	9	6.48107
Yakut	Russia	25	15.60641
Yizu	China	10	3.555
Adygei	Russia	17	6.60288
French	France	28	7.11689
French Basque	France	24	6.3616

North Italian	Italy	13	7.11689
Orcadian	Orkney Islands (Scotland)	15	12.33234
Russian	Russia	25	13.75006
Sardinian	Italy	28	5.69086
Tuscan	Italy	8	6.3616
Bedouin	Israel	46	4.0277
Druze	Israel	42	4.19279
Mozabite	Algeria	29	4.19279
Palestinian	Israel	46	4.19279
NAN Melanesian	Papua New Guinea	11	0.6966
Papuan	Papua New Guinea	17	0.4634
