# Supplementary material for: Genetic adaptation of the human circadian clock to day-length latitudinal variations and relevance for affective disorders
Source: Genome Biol. 2014 Oct 30;15(10):499. doi: 10.1186/s13059-014-0499-7 (PMC4237747; doi:10.1186/s13059-014-0499-7)
Supplement: Additional file 3: — Resampling analysis with SNP number-matched gene sets. [file 13059_2014_499_MOESM3_ESM.pdf]

Additional data file 3.

Resampling analysis with SNP number-matched gene sets

In order to test for the probability that the number of genes (from a given set) with at least one SNP significantly correlated with Δphotoperiod is due to chance, we applied a resampling approach, as described in the main text. Specifically, in those analyses the same number of genes as those in the set being analyzed (study set) was randomly selected from all genes (with at least one SNP genotyped in the HGDP-CEPH panel, n= 15,285) in 10,000 sets (random sets) and the number of genes carrying at least one significant SNP was calculated at each sampling in order to obtain an empirical distribution (hence, an empirical probability). Although we applied Bonferroni correction to p values (i.e. we corrected for the total number of SNPs at each sampling), the number of SNPs in the 5 study sets might bias the results, as long genes, with many variants, may be more likely to carry at least one SNP significantly correlated with Δphotoperiod. To rule out this possibility, we performed additional experiments by sampling genes that match those in the study sets in terms of SNP number. Specifically, we binned the 15,285 genes into classes based on SNP number. Because the distribution of SNP content is strongly skewed towards small values (Supplementary Fig. 1), and due to the extreme heterogeneity of SNP content in large genes, we could not apply a quantile-based binning; rather we applied an empirical criterion based on the requirement that gene classes showing a similar number of SNPs were roughly similar in gene content (Supplementary Fig. 1). The second criterion we applied was that the binning allowed random sets to have a median number of SNPs similar to the SNP number in the study set. In particular, for the 5 study sets we sampled 10,000 SNP-matched random sets (i.e. for each gene in the study set we sampled a random gene from the same gene class in terms of SNP content). We calculated the median number of SNPs for the 10,000 samples and compared it to the number of SNPs in the study set: we decided to accept a maximum difference in SNP number of 2%. Thus, the gene binning was empirically updated until this requirement was satisfied for all sets (Supplementary Tab. 3). Using this approach all gene sets, with the exclusion of genes belonging to melanopsin signaling pathway, were found to display more SNPs correlated with Δphotoperiod than expected (Supplementary Tab. 3). This also applies to the study set deriving from the merging of the 5 independent sets (Supplementary Tab. 3). It is worth mentioning that, on one hand, this approach has the advantage of accounting for SNP content; on the other hand it strongly reduces the independence of random sets, especially for long genes (as is the case of genes involved in melanopsin signaling): as their number is limited they are sampled multiple times, possibly introducing non-independence biases.
