# Supplementary material for: Genetic adaptation of the human circadian clock to day-length latitudinal variations and relevance for affective disorders
Source: Genome Biol. 2014 Oct 30;15(10):499. doi: 10.1186/s13059-014-0499-7 (PMC4237747; doi:10.1186/s13059-014-0499-7)
Supplement: Additional file 4: — Resampling results with SNP-matched random gene sets. [file 13059_2014_499_MOESM4_ESM.pdf]

Additional data file 4. Resampling results with SNP-matched random gene sets.

Gene set	N genes[a]	N SNPs[b]	N significant genes[c]	Median number of sample SNPs[d]	Empirical p value[e]
**Core circadian**	12	175	7	172	0.044
**Circadian hits (RNAi screen)**	223	3863	40	3853	0.021
**Mouse circadian/sleep disturbance**	82	2588	23	2609	0.046
**Mendelian diseases causing sleep disturbance**	11	562	7	554	0.029
**Melanopisn signaling**	13	575	7	583	0.406
**All (merging of the 5 sets)**	341	7763	52	7897	0.040

[a] Number of genes with at least one SNP genotyped in the study set
[b] Number of SNPs in the study set
[c] Number of genes in the study set showing at least one SNP significantly correlated with photoperiod variation
[d] Median number of SNPs in the random sets
[e] Empirical p value calculated as described in the text and Supplementary Text S1.
