# Supplementary material for: Genetic adaptation of the human circadian clock to day-length latitudinal variations and relevance for affective disorders
Source: Genome Biol. 2014 Oct 30;15(10):499. doi: 10.1186/s13059-014-0499-7 (PMC4237747; doi:10.1186/s13059-014-0499-7)

Additional data file 5. Distribution of SNP content. (A) Histogram of SNP content for the 15,285 genes carrying at least one SNP genotyped in the HGDP-CEPH panel. (B) Binning of genes into SNP content classes.

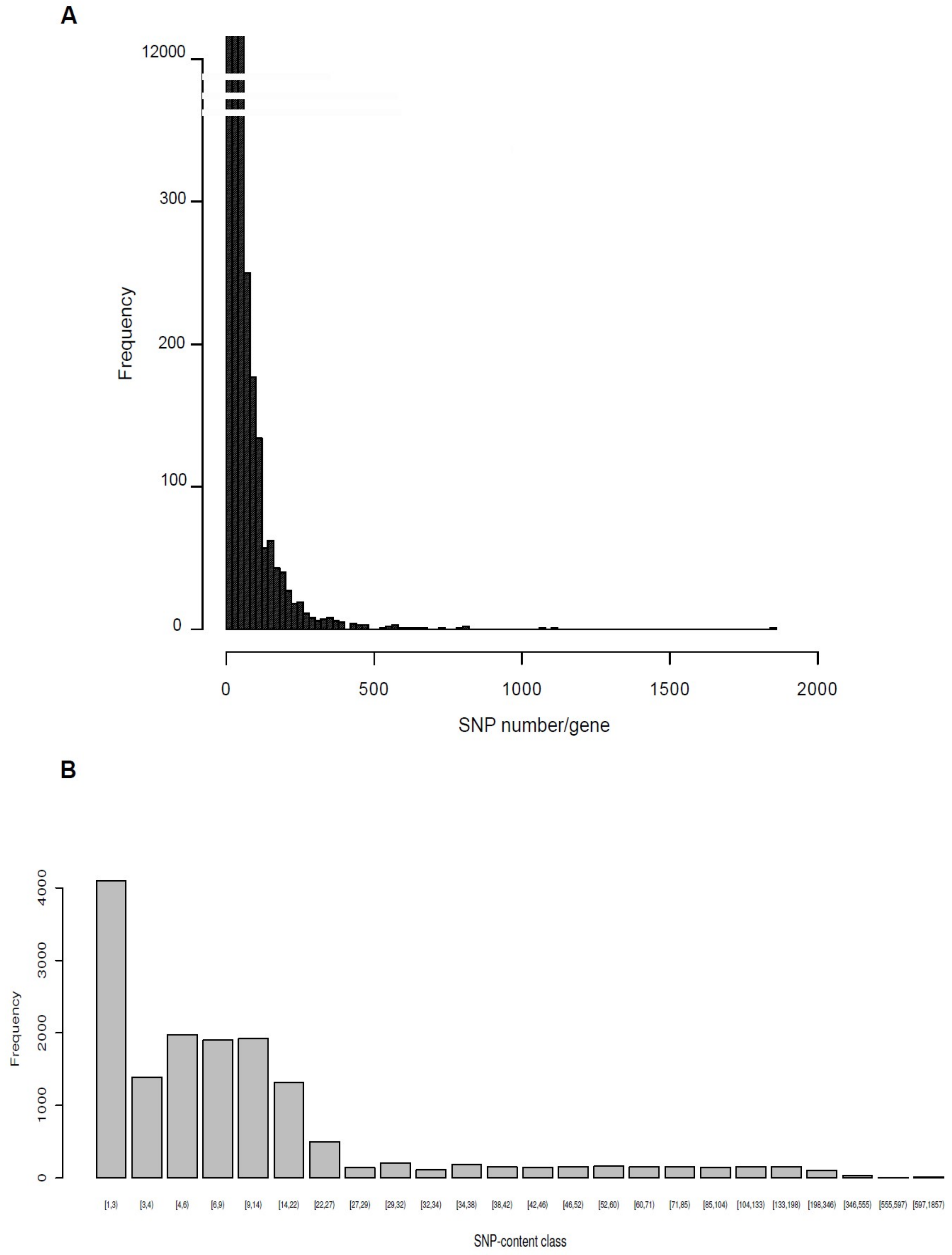

Supplement: Additional file 5: — Distribution of SNP content. [file 13059_2014_499_MOESM5_ESM.pdf]
