# Supplementary material for: Genetic adaptation of the human circadian clock to day-length latitudinal variations and relevance for affective disorders
Source: Genome Biol. 2014 Oct 30;15(10):499. doi: 10.1186/s13059-014-0499-7 (PMC4237747; doi:10.1186/s13059-014-0499-7)
Supplement: Additional file 6: — Resampling analysis with different environmental variables. [file 13059_2014_499_MOESM6_ESM.pdf]

Additional data file 6. Resampling analysis with different environmental variables.

Gene set	N genes[a]	N significant genes[b]	Empirical p value[c]	Contributing genes[d]
**Core circadian**	12	5	0.076 (minimum temperature)	*NPAS2, PER2, ARNTL2, CSNK1D, CSNK1E*
		0	1 (maximum temperature)	-
		2	0.1987 (short wave radiation flux)	*ARNTL2, CSNK1E*
		6	0.0191 (Δtemperature)	*NPAS2, PER2, ARNTL, ARNTL2, CSNK1D, CSNK1E*
**Circadian hits (RNAi screen)**	223	18	0.187 (minimum temperature)	*TPO, ANTXR1, CMTM7, SCHIP1, TBC1D9, PTK2, SH3GL2, ABL1, GPR158, FAM55D, BRCA2, RAB20, CTDP1, HOMER3, SLC8A2, SLC24A3, PWP2, MKL1*
		0	1 (maximum temperature)	-
		2	0.359 (short wave radiation flux)	*SCHIP1, SLC8A2*
		19	0.249 (Δtemperature)	*LZTR2, TPO, ANTXR1, CMTM7, FHIT, TBC1D9, ASB5, JAZF1, SCARA3, POP1, PTK2, SH3GL2, ABL1,GPR158, FAM55D, BRCA2, RAB20, SLC24A3, MKL1*
**Mouse circadian/sleep disturbance**	82	11	0.199 (minimum temperature)	*CAMK2A, EBF2, KCNB2, RORB, TNC, PRKG1, KCNMA1, NCAM1, RORA, NCOR1, APP*
		0	1 (maximum temperature)	-
		1	0.702 (short wave radiation flux)	*DRD3*
		12	0.192 (Δtemperature)	*CAMK2A, CADPS2, EBF2, RORB, TNC, PRKG1, KCNMA1, NCAM1, NOS1, RORA, NCOR1, APP*
**Mendelian diseases causing sleep disturbance**	11	4	0.195 (minimum temperature)	*NRXN1, HDAC4, DHCR7, SHANK3*

		0	1 (maximum temperature)	-
		0	1.000 (short wave radiation flux)	-
		3	0.474 (Δtemperature)	*NRXN1, HDAC4, SHANK3*
**Melanopisn signaling**	13	3	0.538 (minimum temperature)	*PRKCZ, TRPC7, PLCB4*
		0	1 (maximum temperature)	-
		0	1 (short wave radiation flux)	-
		4	0.285 (Δtemperature)	*PRKCZ, PIK3R1, TRPC7, PLCB4*

[a] Number of genes with at least one SNP genotyped in the HGDP-CEPH panel;
[b] Number of genes showing at least one significant SNP;
[c] The empirical p value was calculated as described in the text and methods;
[d] Genes showing at least one significant SNP.
