# Supplementary material for: Genetic adaptation of the human circadian clock to day-length latitudinal variations and relevance for affective disorders
Source: Genome Biol. 2014 Oct 30;15(10):499. doi: 10.1186/s13059-014-0499-7 (PMC4237747; doi:10.1186/s13059-014-0499-7)
Supplement: Additional file 7: — Functional annotation for core circadian genes. [file 13059_2014_499_MOESM7_ESM.pdf]

Additional data file 7. SNP functional annotation for circadian core genes. For each gene the SNP that correlates with Δphotoperiod (underlined) and variants in LD (r^2>0.80) in either CEPH (blue) or CHB+JPT (red) are shown relative to ENCODE annotations.

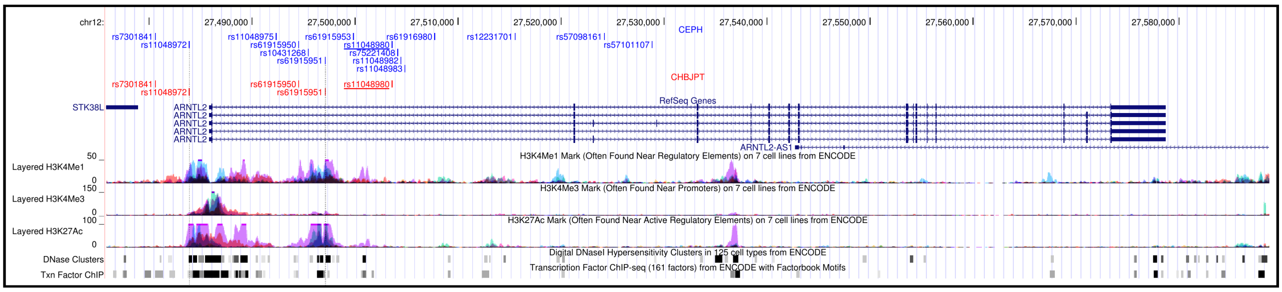

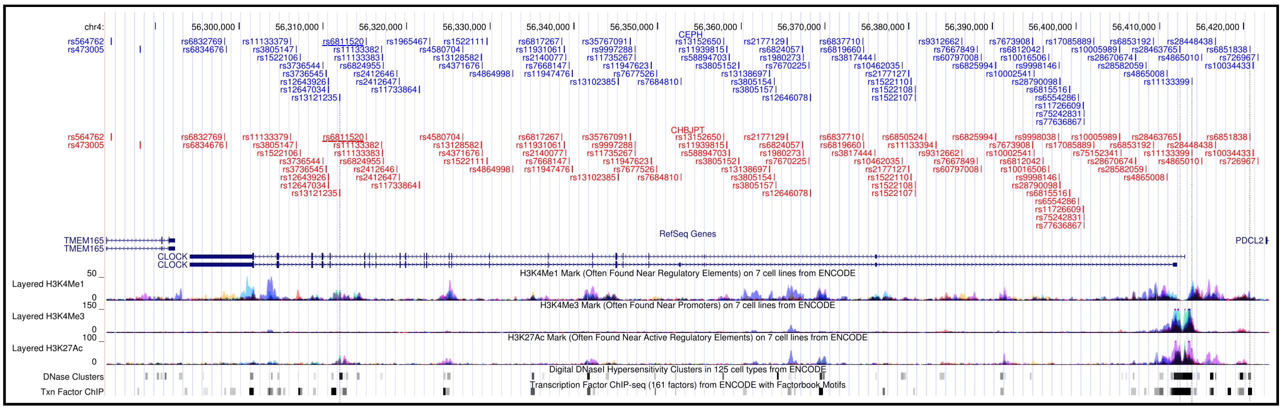

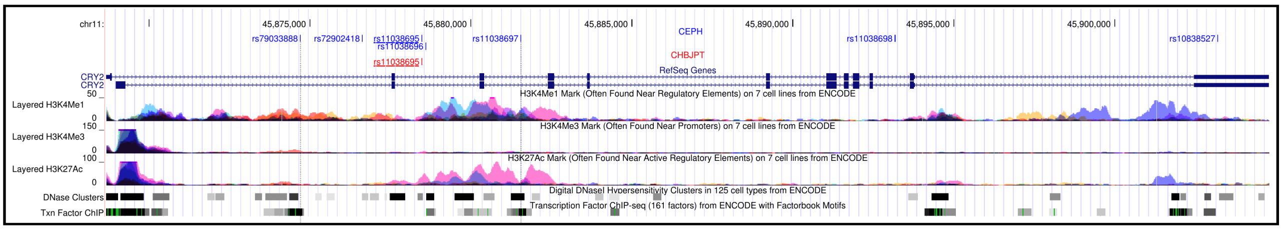

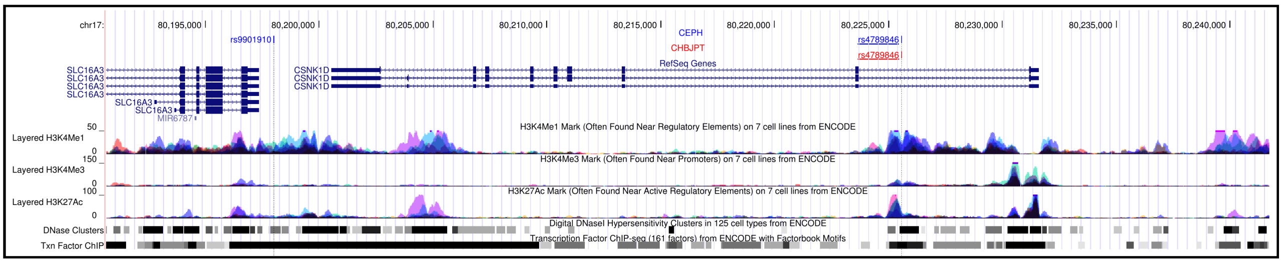

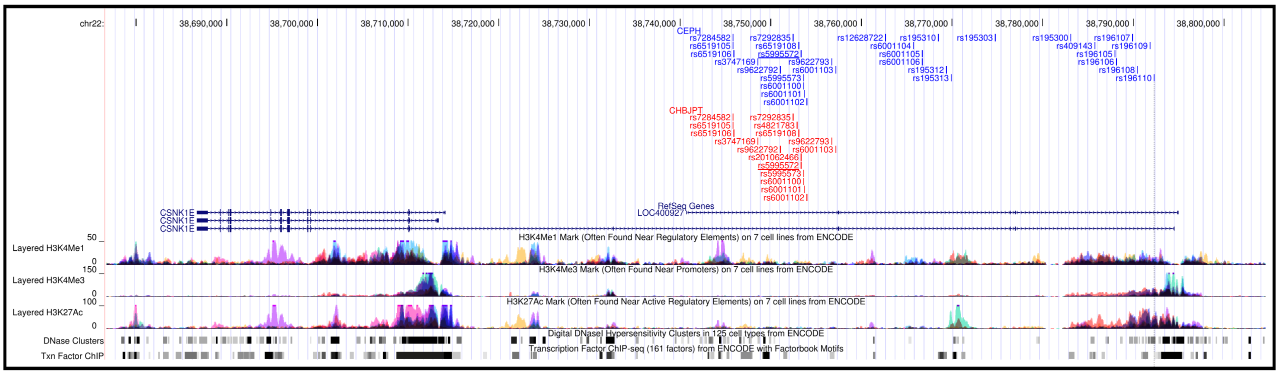
chr22:
38,690,000
38,700,000
38,710,000
38,720,000
38,730,000
38,740,000
38,750,000
38,760,000
38,770,000
38,780,000
38,790,000
38,800,000
CEPH
rs7284582 rs6519105 rs6519106 rs3747169 rs9622792
rs7292835 rs6519108 rs5995572 rs9622793 rs6001103 rs5995573 rs6001100 rs6001101 rs6001102
rs12628722 rs6001104 rs6001105 rs6001106
rs195310 rs195312 rs195313
rs195303
rs195300 rs409143 rs196105 rs196106 rs196108
rs196107 rs196109 rs196110
CHBJPT
rs7284582 rs6519105 rs6519106 rs3747169 rs9622792 rs201062466
rs7292835 rs4821783 rs6519108 rs9622793 rs6001103 rs5995572 rs5995573 rs6001100 rs6001101 rs6001102
RefSeq Genes
LOC400927
CSNK1E
CSNK1E
CSNK1E
Layered H3K4Me1
H3K4Me1 Mark (Often Found Near Regulatory Elements) on 7 cell lines from ENCODE
Layered H3K4Me3
H3K4Me3 Mark (Often Found Near Promoters) on 7 cell lines from ENCODE
Layered H3K27Ac
H3K27Ac Mark (Often Found Near Active Regulatory Elements) on 7 cell lines from ENCODE
DNase Clusters
Digital DNaseI Hypersensitivity Clusters in 125 cell types from ENCODE
Txn Factor ChIP
Transcription Factor ChIP-seq (161 factors) from ENCODE with Factorbook Motifs

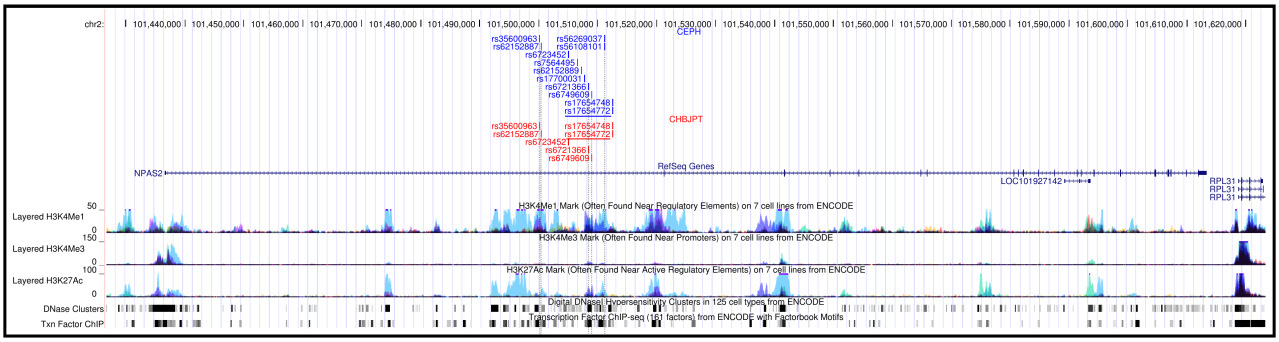
chr2:
101,440,000
101,450,000
101,460,000
101,470,000
101,480,000
101,490,000
101,500,000
101,510,000
101,520,000
101,530,000
101,540,000
101,550,000
101,560,000
101,570,000
101,580,000
101,590,000
101,600,000
101,610,000
101,620,000
CEPH
rs35600963 rs62152887 rs6723452 rs7564495 rs62152889 rs17700031 rs6721366 rs6749609
rs56269037 rs56108101 rs17654748 rs17654772
CHBJPT
rs35600963 rs62152887 rs6723452 rs6721366 rs6749609
rs17654748 rs17654772
RefSeq Genes
NPAS2
LOC101927142
RPL31
RPL31
RPL31
Layered H3K4Me1
H3K4Me1 Mark (Often Found Near Regulatory Elements) on 7 cell lines from ENCODE
Layered H3K4Me3
H3K4Me3 Mark (Often Found Near Promoters) on 7 cell lines from ENCODE
Layered H3K27Ac
H3K27Ac Mark (Often Found Near Active Regulatory Elements) on 7 cell lines from ENCODE
DNase Clusters
Digital DNaseI Hypersensitivity Clusters in 125 cell types from ENCODE
Txn Factor ChIP
Transcription Factor ChIP-seq (161 factors) from ENCODE with Factorbook Motifs

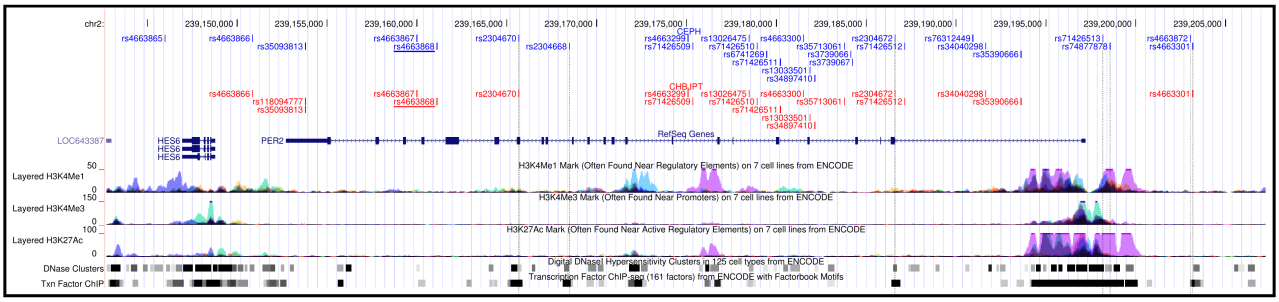
chr2:
239,150,000
239,155,000
239,160,000
239,165,000
239,170,000
239,175,000
239,180,000
239,185,000
239,190,000
239,195,000
239,200,000
239,205,000
CEPH
rs4663865
rs4663866 rs35093813
rs4663867 rs4663868
rs2304670 rs2304668
rs4663299 rs71426509
rs13026475 rs71426510 rs6741269 rs71426511
rs4663300 rs13033501 rs34897410
rs35713061 rs3739066 rs3739067
rs2304672 rs71426512
rs76312449 rs34040298 rs35390666
rs71426513 rs74877878
rs4663872 rs4663301
CHBJPT
rs4663866 rs118094777 rs35093813
rs4663867 rs4663868
rs2304670
rs4663299 rs71426509
rs13026475 rs71426510 rs71426511
rs4663300 rs13033501 rs34897410
rs35713061
rs2304672 rs71426512
rs34040298 rs35390666
rs4663301
RefSeq Genes
LOC643387
HES6
HES6
HES6
PER2
Layered H3K4Me1
H3K4Me1 Mark (Often Found Near Regulatory Elements) on 7 cell lines from ENCODE
Layered H3K4Me3
H3K4Me3 Mark (Often Found Near Promoters) on 7 cell lines from ENCODE
Layered H3K27Ac
H3K27Ac Mark (Often Found Near Active Regulatory Elements) on 7 cell lines from ENCODE
DNase Clusters
Digital DNaseI Hypersensitivity Clusters in 125 cell types from ENCODE
Txn Factor ChIP
Transcription Factor ChIP-seq (161 factors) from ENCODE with Factorbook Motifs
